# Supplementary material for: Barriers and facilitators to palliative care service utilization in Ethiopia: A qualitative systematic review, 2025
Source: PLoS One. 2025 Aug 4;20(8):e0328222. doi: 10.1371/journal.pone.0328222 (PMC12321145; doi:10.1371/journal.pone.0328222)
Supplement: S1 Appendix — (DOCX) [file pone.0328222.s001.docx]

# S1_-_Appendix: Detailed Search Strings

## PubMed/MEDLINE

("Palliative Care"[Mesh] OR "End-of-life care"[Title/Abstract] OR "Hospice"[Title/Abstract])

AND

("Barriers"[Title/Abstract] OR "Facilitators"[Title/Abstract] OR "Challenges"[Title/Abstract] OR "Enablers"[Title/Abstract])

AND

("Qualitative Research"[Mesh] OR "Qualitative"[Title/Abstract])

AND

("Ethiopia"[Mesh] OR "Ethiopia"[Title/Abstract])

## Scopus

TITLE-ABS-KEY("palliative care" OR "end-of-life care" OR "hospice")

AND

TITLE-ABS-KEY("barriers" OR "facilitators" OR "challenges" OR "enablers")

AND

TITLE-ABS-KEY("qualitative")

AND

TITLE-ABS-KEY("Ethiopia")

## Web of Science

TS=("palliative care" OR "end-of-life care" OR "hospice")

AND

TS=("barriers" OR "facilitators" OR "challenges" OR "enablers")

AND

TS=("qualitative")

AND

TS=("Ethiopia")

## CINAHL (via EBSCOhost)

(MH "Palliative Care" OR "end-of-life care" OR "hospice")

AND

("barriers" OR "facilitators" OR "challenges" OR "enablers")

AND

("qualitative")

AND

("Ethiopia")

## Google Scholar

"Palliative care" AND "barriers" AND "qualitative" AND "Ethiopia"

Note: Only the first 200 results sorted by relevance were screened due to Google Scholar's limited search filtering capabilities.
