# Supplementary material for: Barriers and facilitators to palliative care service utilization in Ethiopia: A qualitative systematic review, 2025
Source: PLoS One. 2025 Aug 4;20(8):e0328222. doi: 10.1371/journal.pone.0328222 (PMC12321145; doi:10.1371/journal.pone.0328222)
Supplement: S1 File — (DOCX) [file pone.0328222.s002.docx]

**S1 File -list of identified studies**

| **Study #** | **Citation** | **Title** | **Included?** | **Remark** |
| --- | --- | --- | --- | --- |
| 1 | **Abate et al. BMC Palliative Care**  **(2023) 22:57,** https://doi.org/10.1186/s12904-023-01181-w | Barrier analysis for continuity of palliative care  from health facility to household among adult  cancer patients in Addis Ababa, Ethiopia | Yes | Included |
| 2 | **Aregay et al. BMC Palliative Care**  **(2023) 22:156**  **https://doi.org/10.1186/s12904-023-01283-5** | Palliative care in Ethiopia’s rural &regional health care settings: a qualitative study enabling factors& implementation challenges | Yes | Included |
| 3 | Atsede Aregay, Margaret O'Connor, Jill Stow, Nicola Ayers, and Susan Lee,2023  https://doi.org/10.1177/26323524231198542  DOI: 10.1177/ 26323524231198542 | Perceived policy-related barriers to palliative care implementation: a qualitative study | Yes | Included |
| 4 | **Aregay A, O’Connor M,**  **Stow J, Ayers N, Lee S (2024) Measuring and exploring the barriers to translating palliative care knowledge into clinical practice in rural and regional health-care settings. Palliative and Supportive Care 22(6), 1605–1614.**  **https://doi.org/10.1017/S1478951523000755** | Measuring and exploring the barriers to  translating palliative care knowledge into  clinical practice in rural and regional  health-care settings | Yes | Included |
| 5 | Kaba M, de Fouw M, Deribe KS, Abathun E, Peters AAW, Beltman JJ (2021) Palliative care needs and preferences of female patients and their caregivers in Ethiopia: A rapid program evaluation in Addis Ababa and Sidama zone. PLoS ONE 16(4): e0248738. https://doi.org/10.1371/journal. pone.0248738 | Palliative care needs and preferences of  female patients and their caregivers in  Ethiopia: A rapid program evaluation in Addis  AbabaandSidamazone | Yes | Included |
| 6 | Endalew Hailu Negasa, Sarie Petronella Human & Ameyu Godesso Roro To cite this article: Endalew Hailu Negasa, Sarie Petronella Human & Ameyu Godesso Roro (2023) Challenges in Palliative Care Provision in Ethiopia: An Exploratory Qualitative Study, Journal of Pain Research, 3405-3415, DOI: 10.2147/JPR.S415866 To link to this article: https://doi.org/10.2147/JPR.S415866 | Challenges in Palliative Care Provision in Ethiopia: An Exploratory Qualitative Study | Yes | Included |
| 7 | Eleanor Anderson Reid, MD, MSc, DTM&H,1 Esayas Kebede Gudina, MD, DTM&H, PhD,2 Nicola Ayers, PhD, MSc, BSc (Hons), RGN,3,4 Wondimagegnu Tigineh, MD,5 and Yoseph Mamo Azmera, JOURNAL OF PALLIATIVE MEDICINE Volume 21, Number 5, 2018 a Mary Ann Liebert, Inc. DOI: 10.1089/jpm.2017.0419 | Caring for Life-Limiting Illness in Ethiopia: A Mixed-Methods Assessment of Outpatient Palliative Care Needs | No | Excluded by tittle ,abstract and methodology |
| 8 | Atalay Mulu Fentie, Anteneh Belete & Muluken Nigatu Selam To cite this article: Atalay Mulu Fentie, Anteneh Belete & Muluken Nigatu Selam (2023) Challenges of Access to Oral Morphine Medicine: Palliative Care at a Crossroads for Cancer Patients in Ethiopia, Journal of Pain Research, 1829-1833, DOI: 10.2147/JPR.S410944 To link to this article: https://doi.org/10.2147/JPR.S410944 | Challenges of Access to Oral Morphine Medicine: Palliative Care at a Crossroads for Cancer Patients in Ethiopia | No | Excluded by tittle and abstract |
| 9 | Reid EA, Abathun E, Diribi J, et al. BMJ Supportive & Palliative Care Epub ahead of print: [please include Day Month Year]. doi:10.1136/ spare-2022-003996 | Early palliative care in newly diagnosed cancer in Ethiopia: feasibility randomized controlled trial and cost analysis | No | Excluded by methodology. |
| 10 | Kaba M, de Fouw M, Deribe KS, Abathun E, Peters AAW, Beltman JJ (2021) Palliative care needs and preferences of female patients and their caregivers in Ethiopia: A rapid program evaluation in Addis Ababa and Sidama zone. PLoS ONE 16(4): e0248738. https://doi.org/10.1371/journal. pone.0248738 | Palliative care needs and preferences of female patients and their caregivers in Ethiopia: A rapid program evaluation in Addis Ababa and Sidama zone | No | Excluded by title and abstract |
| 11 | Muday Beneberu1, Getachew Teshale1,2*, Kaleb Assegid Demissie1, Endalkachew Dellie1, Melak Jejaw1 and Asmamaw Atnafu, BMC Palliative Care (2025) 24:57 https://doi.org/10.1186/s12904-025-01694-6 | Patient-centeredness and determinant factors of palliative care service for adult cancer patients in public hospitals of Addis Ababa, Ethiopia, 2024: cross-sectional mixed method study | No | Excluded by methodology |
| 12 | Yoseph Mamo, Anteneh Habte, Nardos W/Giorgis1, Aynalem Abreha3, Nicola Ayers4, Ephrem Abathun1, Eleanor Reid5, Mirgissa Kaba Ethiop. J. Health Dev. 2020; 34(4):310-312]  https://www.ajol.info/index.php/ejhd/article/view/203468/191888 | The evolution of hospice and palliative care in Ethiopia: From historic milestones to future directions | No | Excluded by methodology. |

References

Included article

1. Kaba M, de Fouw M, Deribe KS, Abathun E, Peters AAW, Beltman JJ. Palliative care needs and preferences of female patients and their caregivers in Ethiopia: A rapid program evaluation in Addis Ababa and Sidama zone. PLoS One. 2021;16(4):e0248738.

2. Negasa E, Human SP, Roro A. Challenges in Palliative Care Provision in Ethiopia: An Exploratory Qualitative Study. JPR. 2023 Oct;Volume 16:3405–15.

3. Abate Y, Solomon K, Azmera YM, de Fouw M, Kaba M. Barrier analysis for continuity of palliative care from health facility to household among adult cancer patients in Addis Ababa, Ethiopia. BMC Palliat Care. 2023 May 12;22(1):57.

4. Aregay A, O’Connor M, Stow J, Ayers N, Lee S. Palliative care in Ethiopia’s rural and regional health care settings: a qualitative study of enabling factors and implementation challenges. BMC Palliat Care. 2023 Oct 17;22(1):156.

5. Aregay A, O’Connor M, Stow J, Ayers N, Lee S. Perceived policy-related barriers to palliative care implementation: a qualitative descriptive study. Palliat Care Soc Pract. 2023;17:26323524231198542.

6. Aregay A, O’Connor M, Stow J, Ayers N, Lee S. Measuring and exploring the barriers to translating palliative care knowledge into clinical practice in rural and regional health-care settings. Palliative & Supportive Care. 2024;22(6):1605–14.

**Excluded article**

7. Endalew Hailu Negasa, Sarie Petronella Human & Ameyu Godesso Roro To cite this article: Endalew Hailu Negasa, Sarie Petronella Human & Ameyu Godesso Roro (2023) Challenges in Palliative Care Provision in Ethiopia: An Exploratory Qualitative Study, Journal of Pain Research, 3405-3415, DOI: 10.2147/JPR.S415866 To link to this article: https://doi.org/10.2147/JPR.S415866

8.Eleanor Anderson Reid, MD, MSc, DTM&H,1 Esayas Kebede Gudina, MD, DTM&H, PhD,2 Nicola Ayers, PhD, MSc, BSc (Hons), RGN,3,4 Wondimagegnu Tigineh, MD,5 and Yoseph Mamo Azmera, JOURNAL OF PALLIATIVE MEDICINE Volume 21, Number 5, 2018 a Mary Ann Liebert, Inc. DOI: 10.1089/jpm.2017.0419

9.Atalay Mulu Fentie, Anteneh Belete & Muluken Nigatu Selam To cite this article: Atalay Mulu Fentie, Anteneh Belete & Muluken Nigatu Selam (2023) Challenges of Access to Oral Morphine Medicine: Palliative Care at a Crossroads for Cancer Patients in Ethiopia, Journal of Pain Research, 1829-1833, DOI: 10.2147/JPR.S410944 To link to this article: https://doi.org/10.2147/JPR.S410944

10.Reid EA, Abathun E, Diribi J, et al. BMJ Supportive & Palliative Care Epub ahead of print: [please include Day Month Year]. doi:10.1136/ spare-2022-003996

11.Kaba M, de Fouw M, Deribe KS, Abathun E, Peters AAW, Beltman JJ (2021) Palliative care needs and preferences of female patients and their caregivers in Ethiopia: A rapid program evaluation in Addis Ababa and Sidama zone. PLoS ONE 16(4): e0248738. https://doi.org/10.1371/journal. pone.0248738

12.Muday Beneberu1, Getachew Teshale1,2*, Kaleb Assegid Demissie1, Endalkachew Dellie1, Melak Jejaw1 and Asmamaw Atnafu, BMC Palliative Care (2025) 24:57 https://doi.org/10.1186/s12904-025-01694-6

13.Yoseph Mamo, Anteneh Habte, Nardos W/Giorgis1, Aynalem Abreha3, Nicola Ayers4, Ephrem Abathun1, Eleanor Reid5, Mirgissa Kaba Ethiop. J. Health Dev. 2020; 34(4):310-312]

https://www.ajol.info/index.php/ejhd/article/view/203468/191888
