# Supplementary material for: Barriers and facilitators to palliative care service utilization in Ethiopia: A qualitative systematic review, 2025
Source: PLoS One. 2025 Aug 4;20(8):e0328222. doi: 10.1371/journal.pone.0328222 (PMC12321145; doi:10.1371/journal.pone.0328222)
Supplement: Attachment — Submitted filename: PONE-D-25-20158_reviewer.pdf [file pone.0328222.s003.pdf]

PLOS ONE

Barriers and Facilitators to Palliative Care Utilization in Ethiopia: A Qualitative Systematic Review, 2025

--Manuscript Draft--

**Manuscript Number:**	PONE-D-25-20158
**Article Type:**	Research Article
**Full Title:**	Barriers and Facilitators to Palliative Care Utilization in Ethiopia: A Qualitative Systematic Review, 2025
**Short Title:**	Barriers and Facilitators to Palliative Care Utilization in Ethiopia
**Corresponding Author:**	Sadik Abdulwehab, BSc,MSC in Adult Health Nursing, PhD fellow<br>Wollega University<br>Nekemte, Oromia ETHIOPIA
**Keywords:**	Palliative care; Utilization; Barriers; Facilitators; Ethiopia
**Abstract:**	Introduction<br><br>Palliative care is a crucial part of end stage disease management, but its utilization remains low in low- and middle-income countries like Ethiopia. This is due to various systemic, social, and policy barriers. Understanding these contextual factors is crucial for developing effective interventions and policy frameworks. This study aimed to explore and synthesize the barriers and facilitators to palliative care utilization in Ethiopia using a qualitative systematic review approach.<br><br>Methods<br><br>This review followed the PRISMA 2020 and PRISMA-QS guidelines, employing a qualitative systematic design. A comprehensive search was conducted across different databases using tailored keywords and MeSH terms until March 19, 2025. The database was searched for every article published on palliative care services till March 10, 2025, and continued to update until we sent it for publication. The data was extracted from March 11-20 and later analyzed from March 21-30, and the report generation till April 10, 2025. Thematic synthesis was used to analyze findings and the GRADE-CERQual approach was employed to assess the confidence of evidence<br><br>Results<br><br>Six studies met the inclusion criteria, encompassing diverse Ethiopian healthcare settings and stakeholders. Five major barriers were identified: policy and governance gaps, health system challenges, knowledge and training deficits, sociocultural and economic constraints, and poor collaboration. In contrast, five facilitators emerged: strong community and family support, intrinsic healthcare provider motivation, integration of palliative care into education, holistic care models, and stakeholder engagement. High confidence was assigned to four themes, underscoring their significance and applicability.<br><br>Conclusion<br><br>The underutilization of palliative care in Ethiopia stems from intertwined structural, educational, and sociocultural challenges. However, promising facilitators exist that can guide policy reform and intervention design. Addressing these barriers through improved policies, workforce development, and community engagement is imperative for ensuring equitable access to quality palliative care services.
**Order of Authors:**	Sadik Abdulwehab, BSc,MSC in Adult Health Nursing, PhD fellow
	Frezer Kedir, BSc, Msc
**Additional Information:**	
**Question**	**Response**

Powered by Editorial Manager® and ProduXion Manager® from Aries Systems Corporation

**Financial Disclosure**<br><br>Enter a financial disclosure statement that describes the sources of funding for the work included in this submission. Review the submission guidelines for detailed requirements. View published research articles from *PLOS ONE* for specific examples.<br><br>This statement is required for submission and **will appear in the published article** if the submission is accepted. Please make sure it is accurate.<br><br>**Funded studies**<br>Enter a statement with the following details:<br>• Initials of the authors who received each award<br>• Grant numbers awarded to each author<br>• The full name of each funder<br>• URL of each funder website<br>• Did the sponsors or funders play any role in the study design, data collection and analysis, decision to publish, or preparation of the manuscript?<br><br>Did you receive funding for this work?	The author(s) received no specific funding for this work.
**Competing Interests**<br><br>Use the instructions below to enter a competing interest statement for this submission. On behalf of all authors, disclose any competing interests that could be perceived to bias this work—acknowledging all financial support and any other relevant financial or non-financial competing interests.<br><br>This statement is required for submission and **will appear in the published article** if the submission is accepted. Please make sure it is accurate and that any funding sources listed in your Funding Information later in the submission form are also declared in your Financial Disclosure statement.	The authors have declared that no competing interests exist.

Powered by Editorial Manager® and ProduXion Manager® from Aries Systems Corporation

View published research articles from *PLOS ONE* for specific examples.<br><br>**NO authors have competing interests**<br><br>Enter: *The authors have declared that no competing interests exist.*<br><br>**Authors with competing interests**<br><br>Enter competing interest details beginning with this statement:<br><br>*I have read the journal's policy and the authors of this manuscript have the following competing interests: [insert competing interests here]*<br><br>* typeset	
**Ethics Statement**<br><br>Enter an ethics statement for this submission. This statement is required if the study involved:<br><br>• Human participants<br>• Human specimens or tissue<br>• Vertebrate animals or cephalopods<br>• Vertebrate embryos or tissues<br>• Field research<br><br>Write "N/A" if the submission does not require an ethics statement.<br><br>General guidance is provided below. Consult the submission guidelines for detailed instructions. **Make sure that all information entered here is included in the Methods section of the manuscript.**	N/A

Powered by Editorial Manager® and ProduXion Manager® from Aries Systems Corporation

**Format for specific study types**<br><br>**Human Subject Research (involving human participants and/or tissue)**<br>• Give the name of the institutional review board or ethics committee that approved the study<br>• Include the approval number and/or a statement indicating approval of this research<br>• Indicate the form of consent obtained (written/oral) or the reason that consent was not obtained (e.g. the data were analyzed anonymously)<br><br>**Animal Research (involving vertebrate animals, embryos or tissues)**<br>• Provide the name of the Institutional Animal Care and Use Committee (IACUC) or other relevant ethics board that reviewed the study protocol, and indicate whether they approved this research or granted a formal waiver of ethical approval<br>• Include an approval number if one was obtained<br>• If the study involved *non-human primates*, add *additional details* about animal welfare and steps taken to ameliorate suffering<br>• If anesthesia, euthanasia, or any kind of animal sacrifice is part of the study, include briefly which substances and/or methods were applied<br><br>**Field Research**<br><br>Include the following details if this study involves the collection of plant, animal, or other materials from a natural setting:<br>• Field permit number<br>• Name of the institution or relevant body that granted permission	
**Data Availability**<br><br>Authors are required to make all data underlying the findings described fully available, without restriction, and from the time of publication. PLOS allows rare exceptions to address legal and ethical concerns. See the PLOS Data Policy and FAQ for detailed information.	Yes - all data are fully available without restriction

Powered by Editorial Manager® and ProduXion Manager® from Aries Systems Corporation

A Data Availability Statement describing where the data can be found is required at submission. Your answers to this question constitute the Data Availability Statement and **will be published in the article**, if accepted.<br><br>**Important:** Stating 'data available on request from the author' is not sufficient. If your data are only available upon request, select 'No' for the first question and explain your exceptional situation in the text box.<br><br>Do the authors confirm that all data underlying the findings described in their manuscript are fully available without restriction?	
**Describe where the data may be found in full sentences. If you are copying our sample text, replace any instances of XXX with the appropriate details.**<br><br>• If the data are **held or will be held in a public repository**, include URLs, accession numbers or DOIs. If this information will only be available after acceptance, indicate this by ticking the box below. For example: *All XXX files are available from the XXX database (accession number(s) XXX, XXX.).*<br>• If the data are all contained **within the manuscript and/or Supporting Information files**, enter the following: *All relevant data are within the manuscript and its Supporting Information files.*<br>• If neither of these applies but you are able to provide **details of access elsewhere**, with or without limitations, please do so. For example:<br><br>*Data cannot be shared publicly because of [XXX]. Data are available from the XXX Institutional Data Access / Ethics Committee (contact via XXX) for researchers who meet the criteria for access to confidential data.*<br><br>*The data underlying the results presented in the study are available from (include the name of the third party*	All relevant data are within the manuscript and its Supporting Information files

and contact information or URL).<br>• This text is appropriate if the data are owned by a third party and authors do not have permission to share the data.<br><br>* typeset	
Additional data availability information:	

Full manuscript Click here to access/download;Manuscript;Full Manuscript.docx

Title Page

Article Title

Barriers and Facilitators to Palliative Care Utilization in Ethiopia: A Qualitative Systematic Review, 2025

Author Names

Sadik Abdulwehab, BSc, MSc[1]; https://orcid.org/0009-0006-0863-4288

Frezer Kedir BSc, Msc[2] ; https://orcid.org/0009-0000-6613-9441

Author Affiliations

[1] School of Nursing, Wollega University, Oromia, Ethiopia

[2]School of Nursing, Jimma University; Southwest Oromia; Ethiopia

Email address of the authors

sadikabdulwehab@gmail.com and frezerkedir@gmail.com

Corresponding Author Information

[1] School of Nursing, College of Health Science, Wollega University,

P.O. Box: 378, Nekemte, Oromia, Ethiopia

Email; sadikabdulwehab@gmail.com

Phone number +251911738062

Orchid; *https://orcid.org/0009-0006-0863-4288*

Abstract

Introduction: Palliative care is a crucial part of end stage disease management, but its utilization remains low in low- and middle-income countries like Ethiopia. This is due to various systemic, social, and policy barriers. Understanding these contextual factors is crucial for developing effective interventions and policy frameworks. This study aimed to explore and synthesize the barriers and facilitators to palliative care utilization in Ethiopia using a qualitative systematic review approach.

Methods: This review followed the PRISMA 2020 and PRISMA-QS guidelines, employing a qualitative systematic design. A comprehensive search was conducted across different databases using tailored keywords and MeSH terms until March 19, 2025. The database was searched for every article published on palliative care services till March 10, 2025, and continued to update until we sent it for publication. The data was extracted from March 11-20 and later analyzed from March 21-30, and the report generation till April 10, 2025. Thematic synthesis was used to analyze findings and the GRADE-CERQual approach was employed to assess the confidence of evidence

Results: Six studies met the inclusion criteria, encompassing diverse Ethiopian healthcare settings and stakeholders. Five major barriers were identified: policy and governance gaps, health system challenges, knowledge and training deficits, sociocultural and economic constraints, and poor collaboration. In contrast, five facilitators emerged: strong community and family support, intrinsic healthcare provider motivation, integration of palliative care into education, holistic care models, and stakeholder engagement. High confidence was assigned to four themes, underscoring their significance and applicability.

Conclusion: The underutilization of palliative care in Ethiopia stems from intertwined structural, educational, and sociocultural challenges. However, promising facilitators exist that can guide policy reform and intervention design. Addressing these barriers through improved policies, workforce development, and community engagement is imperative for ensuring equitable access to quality palliative care services.

Keywords: Palliative care, Utilization, Barriers, Facilitators, Ethiopia

Highlight of the review

What is Known

- Palliative care is widely underutilized in LMICs like Ethiopia due to systemic and resource-related limitations.
- Common global barriers include weak policies, insufficient training, and poor service integration.
- Cultural norms and financial challenges often drive families to seek informal or home-based care instead of formal services.

What this paper adds to the existing body of knowledge

- This is the first review focused specifically on Ethiopia, providing locally grounded evidence.
- It identifies both challenges and enablers of palliative care, offering a balanced perspective.
- The study highlights the crucial role of community networks, faith-based support, and curriculum integration in overcoming systemic gaps and improving access to palliative care.

Introduction

According to the International Association for Hospice and Palliative Care (IAHPC), Palliative care is defined as active holistic care of individuals across all ages with serious health-related suffering due to severe illness, especially of those near the end of life aimed to improve the quality of life of patients, their families, and their caregivers (1,2). Palliative care service is not limited to end-of-life scenarios but applies to anyone experiencing profound health-related suffering, regardless of age or disease stage(3). The approach includes prevention, early identification, comprehensive assessment, and management of physical, psychological, social, and spiritual issues(4).

Globally, palliative care remains significantly underutilized as the World Health Organization (WHO) shows, that only about 1 in 10 people who require palliative care receive it. This gap is particularly pronounced in low- and middle-income countries (LMICs), where approximately 78% of those in need reside (5). In 2017, seven million patients worldwide received specialized palliative care services, but this only accounts for a fraction of the global need, which includes over 56.8 million individuals annually as 69% of patients were in high-income countries, 27% in upper-middle-income countries, and 4% in lower-middle countries (6).

Despite increasing recognition of its importance, palliative care remains vastly underutilized worldwide, posing a significant public health challenge(7). Globally, over 56.8 million people require palliative care annually, yet only about 14% receive it, leaving the vast majority without essential relief from pain, distress, and psychosocial suffering (8). This care gap is particularly acute in low- and middle-income countries (LMICs), where 78% of the global need exists but access to services is minimal or non-existent. Many countries still lack national policies, essential medications like opioids, trained professionals, or community-based palliative care models (9).

The consequences are profound as millions die each year in severe pain and with diminished quality of life, highlighting not only a health system failure but a deep ethical and human rights concern. Bridging this gap requires an urgent international commitment to integrating palliative care into universal health coverage and strengthening healthcare systems with culturally and contextually appropriate solutions (10).

Palliative care utilization is severely constrained by a range of barriers and limiting factors, particularly in low- and middle-income countries. Key barriers include a lack of trained healthcare

providers, limited availability of essential medications (especially opioids for pain control), and weak integration into national health systems (9). Additionally, cultural misconceptions, poor awareness among patients and providers, and social stigma and death further hinder uptake (7). Structural issues, such as inadequate funding, geographic inaccessibility, and absence of national policies, compound the problem. These barriers not only limit access to palliative care services but also contribute to widespread suffering and poor quality of life at the end of life(11).

The lack of access to palliative care services significantly impacts patients, families, and health systems worldwide, leading to pain, psychological distress, spiritual suffering, and loss of dignity, as well as increased hospital admissions, higher healthcare costs, and overuse of aggressive treatments(7,9). Palliative care delivery is a global issue with key gaps such as lack of national policies, shortage of trained providers, restricted opioid access, poor integration, limited funding, and insufficient community awareness, contributing to a global crisis (5,7,9).
Palliative care services in Ethiopia are in their early stages, with limited access and a high concentration in urban centers, despite the increasing prevalence of chronic conditions, leaving rural populations underserved (1). Ethiopia's palliative care policy is inadequate, with limited integration into primary care, challenges like limited healthcare worker training, cultural taboos, and limited drug availability, emphasizing the urgent need for prioritization in healthcare(2–4).
This qualitative systematic review aims to explore the barriers and facilitators to palliative care utilization in Ethiopia by synthesizing findings from multiple primary studies. By integrating the evidence, the review seeks to provide a comprehensive understanding of the factors contributing to the underutilization of palliative care in this population and highlight facilitators. By identifying and connecting these factors across studies, the findings of this review are expected to guide future policy development, inform targeted interventions, and promote the integration of palliative care into Ethiopia's national healthcare framework, in alignment with global palliative care priorities and WHO recommendations.

Methods

Study Design

This study employed a qualitative systematic review design to synthesize existing research evidence on the barriers and facilitators to palliative care utilization in Ethiopia. The review process was guided by the Preferred Reporting Items for Systematic Reviews and Meta-Analyses

(PRISMA) 2020 guidelines (11) and the PRISMA Extension for Qualitative Evidence Synthesis (PRISMA-QS) (12), ensuring transparency and methodological rigor throughout the process.

Aim of the study

This review aimed to provide a contextualized understanding of the factors influencing palliative care access and use in the Ethiopian healthcare system by consolidating qualitative findings from primary studies.

Review Protocol

To promote reproducibility and transparency, a review protocol was developed before the conduct of the study. This protocol outlined key elements including the research objectives, eligibility criteria, search strategy, data extraction procedures, quality appraisal framework, and synthesis approach. The protocol was developed in alignment with the Joanna Briggs Institute (JBI) Manual for Evidence Synthesis (13), which provides comprehensive guidance for conducting qualitative systematic reviews. To ensure accountability and reduce the risk of bias, the protocol was registered with registration number CRD420251027739 on April 6, 2025, serving as a formal commitment to the planned methodology.

Search Strategy

A comprehensive and systematic search of literature was conducted across major electronic databases including PubMed/MEDLINE, Scopus, Web of Science, CINAHL, and Google Scholar. The search strategy was designed to capture all relevant studies published up to April 10 and updated till we sent the paper for publication, and it involved the use of both free-text keywords and Medical Subject Headings (MeSH) to enhance sensitivity and specificity. Boolean operators were employed to combine search terms, including: ("Palliative Care" OR "End-of-life care" OR "Hospice") AND ("Barriers" OR "Facilitators" OR "Challenges" OR "Enablers") AND ("Qualitative") AND ("Ethiopia"). Additionally, backward citation tracking of reference lists from included studies was performed to identify any further eligible studies missed in the initial database search.

Inclusion and exclusion criteria

Eligibility criteria were structured using the SPIDER tool, which is particularly appropriate for identifying studies in qualitative syntheses. The sample (S) included patients, caregivers, and healthcare professionals involved in palliative care delivery in Ethiopia. The phenomenon of interest (PI) focused on the utilization of palliative care services, with an emphasis on identifying

both barriers and facilitators. Eligible studies employed qualitative designs (D), including in-depth interviews, focus groups, ethnographies, or other narrative approaches. Evaluation (E) centered on participants' lived experiences, perceptions, and meanings surrounding access to palliative care. Only primary research articles that were qualitative or mixed-methods with clearly separable qualitative findings (R) were included. Studies were eligible if they were conducted in Ethiopia, and focused on palliative care. Although we searched grey literature or unpublished sources. The search was not restricted by date or language to enhance inclusivity and reduce the risk of missing relevant qualitative studies. Excluded were purely quantitative studies, reviews, opinion pieces, editorials, conference abstracts, and any research conducted outside Ethiopia.

Search and Screening

All search results were imported into Zotero to facilitate reference management and removal of duplicates. Titles and abstracts were independently screened by two reviewers based on the predetermined inclusion criteria. Full-text articles that met initial criteria were then retrieved and assessed for final eligibility. Discrepancies between reviewers were discussed and resolved by agreement. The study selection process was documented using a PRISMA 2020 flow diagram, which clearly illustrated the number of studies screened, excluded, and included, along with reasons for exclusion at the full-text stage (Figure 1).

The database was searched for every article published on palliative care services till March 10, 2025, and continued to update until we sent it for publication. The data was extracted from March 11-20 and later analyzed from March 21-30, and the report generation till April 10, 2025.

Quality Appraisal

To assess the methodological robustness of the included studies, the Critical Appraisal Skills Programme qualitative checklist (14) was applied. This tool evaluates ten domains including clarity of the research aim, appropriateness of design, recruitment strategies, data collection methods, ethical considerations, reflexivity, rigor in analysis, and overall value. Two reviewers independently appraised the quality of each study and recorded their judgments. While no study was excluded based solely on quality, the appraisal outcomes were used to interpret the strength and trustworthiness of the synthesized findings.

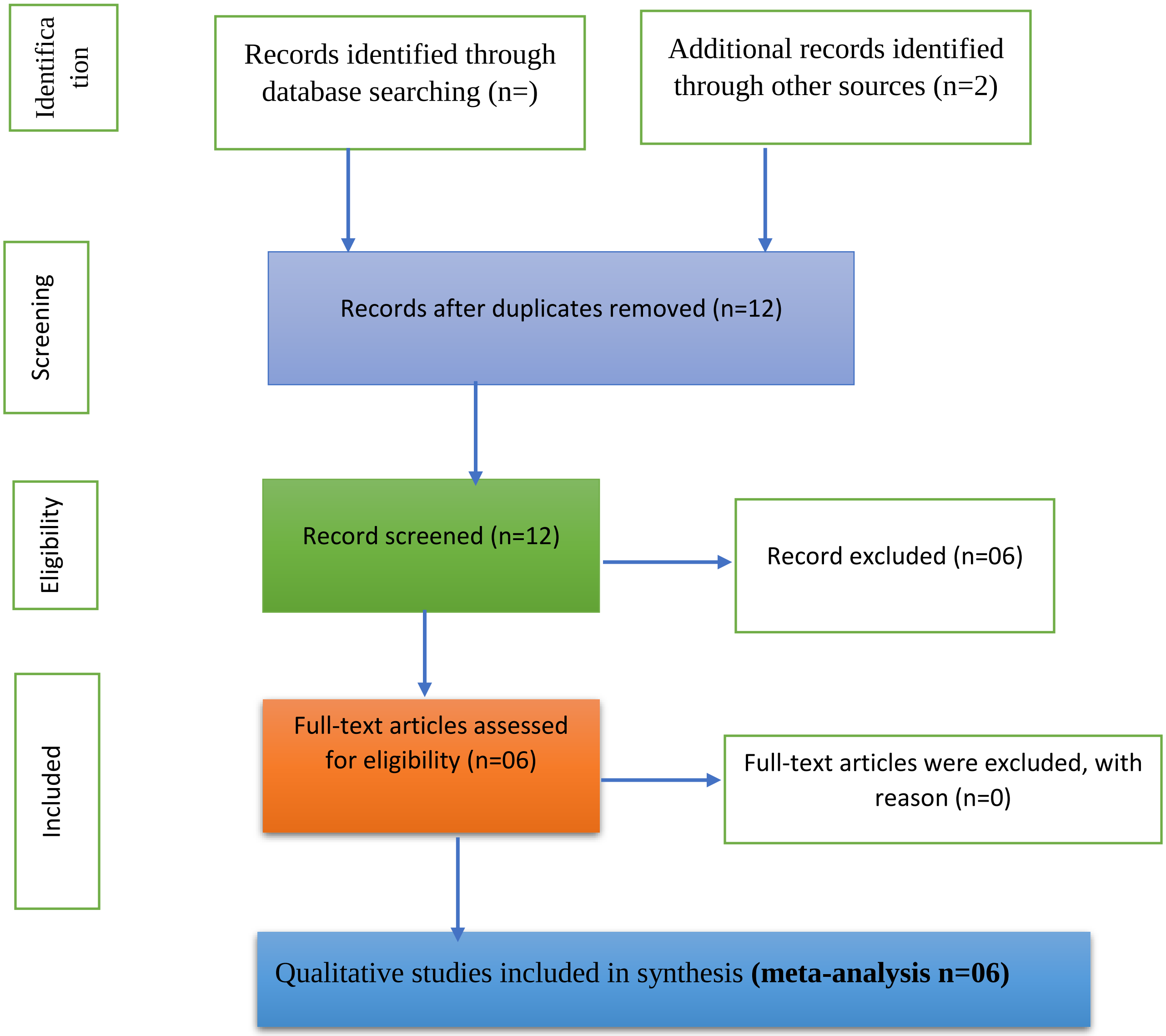

Figure. 1. *Flow chart diagram of PRISMA 2020 checklist describing the selection of studies for the systematic review and meta-analysis on Palliative care in Ethiopia, 2025*

Data Extraction

A standardized and piloted data extraction form was developed to ensure consistency in collecting relevant study characteristics and findings. Information extracted included author(s) and year of publication, study setting, research objectives, study design, participant characteristics, data collection and analysis methods, key findings related to barriers and facilitators, and illustrative participant quotes. Two reviewers performed the extraction independently, and discrepancies were

resolved through dialogue and consensus, ensuring the reliability and accuracy of the data extracted for synthesis.

Data Synthesis

Thematic synthesis, as described by Thomas and Harden (15), was employed to integrate findings from the included studies. This method involved a three-step process: line-by-line coding of the primary data (both participant quotes and authors' interpretations), grouping of codes to generate descriptive themes, and the development of higher-order analytical themes that offered deeper insight into the review question. The synthesis was iterative and collaborative, with co-authors engaging in critical reflection to refine themes, enhance credibility, and ensure resonance with the Ethiopian sociocultural and healthcare context.

Assessment of Confidence in the Evidence

To evaluate the confidence in each synthesized theme, the GRADE-CERQual (Confidence in the Evidence from Reviews of Qualitative Research) (16) approach was applied. Each key finding was assessed across four domains: methodological limitations of the contributing studies, coherence of the data supporting the finding, adequacy of the data in terms of richness and quantity, and relevance to the review question. Confidence levels were assigned as high, moderate, low, or very low, and rationales for each judgment were documented. This approach provided a transparent appraisal of the strength of the evidence underpinning the review's conclusions.

Results

Study selection

A total of 76 records were retrieved from the database search and 02 additional results through other sources, of which 78 unique records were obtained. After screening titles and abstracts, 12 articles remained for full-text screening. Six of these studies met the eligibility criteria. Figure 1 presents the screening process of studies included in this review.

The studies reviewed span diverse settings within Ethiopia, offering a comprehensive view of palliative care delivery across the country (17–22). Abate et al. (17) focused on both health facilities and households to examine barriers to the continuity of care. Aregay et al. (2023a) (19) and Aregay et al. (2024) (21) conducted their studies in rural and regional health facilities, with the latter extending to primary, general, and comprehensive hospitals. Aregay et al. (2023b) (20) shifted focus to the policy level, involving various national and regional health offices. Kaba et al. (22) included urban areas of Addis Ababa and Yirgalem where three palliative care programs were

evaluated. Similarly, Negasa et al. (18) explored challenges from both central (Addis Ababa) and regional (Jimma Zone) settings, engaging with a wide range of stakeholders and health workers.

Each study had distinct but complementary objectives. Abate et al. (17) aimed to understand the barriers to ensuring continuity of palliative care from health facilities to the household level. Aregay et al. (2023a) (19) explored both the enabling and limiting factors for palliative care service delivery in rural contexts, while Aregay et al. (2023b) (20) focused on identifying national policy barriers that hinder the implementation of palliative care. Aregay et al. (21) investigated the translation gap between palliative care knowledge and clinical practice. Kaba et al. (22) examined the needs and preferences of female patients and their caregivers within existing palliative care programs. Lastly, Negasa et al. (18) explored systemic, provider, patient, and partnership-related challenges facing palliative care in Ethiopia.

All the studies employed qualitative designs (17–20,22), except Aregay et al.(21), which used a mixed-method approach that combined a quantitative nurse survey with in-depth interviews and was included in this review due to its robust and delineated qualitative component. Only the qualitative findings relevant to barriers and facilitators of palliative care utilization were extracted and synthesized for this review, in line with the inclusion criteria. All studies utilized interviews as their primary data collection method (17–22). Most studies used thematic analysis to interpret data, with ATLAS. Ti was employed in studies by Abate et al. (17) and Negasa et al.(18), and NVivo was used in Aregay et al.(21). The studies varied in methodological approach from rapid program evaluations(22) to key informant interviews(20) and exploratory qualitative designs(18).

Across the included studies, participants represented a diverse group involved in palliative care. Abate et al.(17), included mainly health extension workers and caregivers. Aregay et al. (2023a)(19) and Aregay et al.(21) engaged clinical and managerial health professionals, while Aregay et al. (2023b)(20) focused on high-level policy and program decision-makers. Kaba et al. (22)offered a broader perspective by involving patients, primary and voluntary caregivers, and institutional stakeholders. Negasa et al.(18) included both policy actors and frontline health workers, supported by focus group discussions with nurses from chronic care wards.

Methodological quality of included studies

The methodological quality of the included studies was appraised using the CASP checklist. All studies had a clear statement of research aims, employed appropriate qualitative methodologies, and utilized research designs aligned with their objectives. Ethical issues were mentioned or implied in some studies, and all presented clear findings relevant to the research questions. Most studies (83%) applied suitable recruitment strategies and conducted data collection methods that addressed their research issues effectively (17–20,22). Similarly, the majority demonstrated sufficiently rigorous data analysis (100%) (17–22). However, only two studies (33%) adequately discussed or reflected on the relationship between researchers and participants(21,22), indicating an area for improvement in reflexivity reporting (Table 2).

Synthesis

The qualitative synthesis of the included studies revealed two overarching categories**:** barriers and facilitators to the implementation and delivery of palliative care in Ethiopia. Under barriers, five major themes emerged: policy and governance gaps, health system challenges, knowledge and training deficits, sociocultural and economic barriers, and collaboration challenges. These themes reflect persistent structural, educational, and systemic issues that hinder effective palliative care. Illustrative quotes reveal the absence of national policies, insufficient training, weak infrastructure, cultural preferences for home-based death, and a lack of coordinated efforts among stakeholders.

In contrast, five key themes were identified as facilitators: community and family support, healthcare provider motivation, education and curriculum inclusion, holistic care models, and stakeholder involvement. These enablers underscore the value of grassroots efforts, personal commitment among providers, integration of palliative care into higher education, and supportive roles played by NGOs and faith-based organizations. The themes, accompanying quotes, and contributing studies are summarized in Table 3.

Policy and Governance Gaps

The theme of policy and governance gaps emerged prominently across the included studies and consisted of four interrelated subthemes: absence of national policy, limited stakeholder engagement, lack of a dedicated budget, and weak leadership and implementation frameworks. The absence of an articulated national policy for palliative care was cited in multiple studies(17,20). This policy void was seen as a foundational barrier, undermining the institutional prioritization of palliative care. In several cases, respondents emphasized that palliative care was

not integrated into national or regional performance frameworks, reducing its visibility within public health agendas: “Palliative care is not included in performance plans, so staff don’t consider it a priority”(17).

Stakeholder engagement was also found to be minimal, particularly at the community level, where policies often failed to address care continuity from health facilities to homes. Aregay et al. (20) highlighted that the absence of community representation in strategic planning led to neglect in rural and underserved areas. Budget limitations were closely linked to this strategic neglect, with multiple studies noting the absence of a defined financial line for palliative care services within governmental structures: “There is no budget line for palliative care in the ministry’s structure” (17). This fiscal exclusion perpetuated resource constraints and further discouraged the formal development of service models. Lastly, leadership and implementation barriers were widely reported. A consistent concern across studies was the lack of committed leadership and clear implementation frameworks to support even the limited palliative care services that exist (20). Collectively, these subthemes reflect a broader systemic marginalization of palliative care within the health governance landscape in Ethiopia, contributing to fragmented service delivery and inequitable access.

Policy and Governance Gaps

The theme of policy and governance gaps comprised four subthemes: absence of a national policy, limited stakeholder engagement, lack of a dedicated budget, and weak leadership and implementation frameworks. Multiple studies reported that palliative care is not integrated into national or regional strategic plans, which reduces its institutional visibility and prioritization (17,20). For instance, participants noted, “Palliative care is not included in performance plans, so staff don’t consider it a priority” (17). Additionally, community-level needs are often excluded from policy discussions, reflecting broader neglect in health governance(20). The lack of a dedicated budget further exacerbates the issue, making it difficult to operationalize services: “There is no budget line for palliative care in the ministry’s structure” (17). These limitations are compounded by weak leadership and insufficient commitment to implement guidelines effectively.

Health System Challenges

Health system challenges emerged as another critical theme and included subthemes such as a shortage of trained staff, poor service integration, weak referral systems, urban-rural service disparities, and inadequate infrastructure. Staff often reported being overburdened and

undertrained, which undermines care quality: “We are overloaded and lack training, but we try to help when possible” (19). Unstructured and poorly coordinated care models were prevalent, as noted by a participant: “There is no organized palliative care service; we just do what we can” (19). Services were highly centralized, disadvantaging rural populations (19). Furthermore, shortages in medicines and medical infrastructure were identified as ongoing challenges (18).

Knowledge and Training Deficits

This theme included limited provider awareness, absence of palliative care in educational curricula, and inadequate training methods. Healthcare professionals expressed uncertainty in delivering care due to insufficient knowledge: “We do not have detailed information on how to provide care for chronically ill patients”(21). Participants also indicated that training programs did not adequately prepare them for real-world scenarios: “The traditional lecture-based system… does not work” (21). The lack of curriculum content in undergraduate programs further contributes to this knowledge gap (18).

Sociocultural and Economic Barriers

This theme encompassed cultural preferences, financial hardship, and inadequate psychosocial and spiritual support. Some participants highlighted a cultural preference for dying at home, which limits formal care utilization: “Patients prefer to die at home... believing it’s more respectful”(18). Financial barriers were equally pronounced, with many families unable to sustain care: “Discontinuation of care due to cost is common”(18). Emotional and spiritual needs were frequently unmet: “We address pain, but there’s no spiritual or economic support”(22).

Collaboration Challenges

Weak coordination between government and non-governmental actors was a recurrent issue. For instance, limited partnerships between the public health sector and NGOs were found to hinder service integration: “Limited partnership between government and NGOs impedes service integration” (18). This lack of coordinated effort reduces resource pooling, knowledge exchange, and scalability of care models.

Theme formulated as Facilitators to Palliative Care

Community and Family Support

This facilitating theme included community-based involvement, religious networks, and informal caregiving systems. Participants frequently credited community support for sustaining care delivery in the absence of formal mechanisms: “Community support helps us a lot, especially when

professionals don't show up" (17). Religious and family structures often acted as informal care systems, particularly in rural and underserved settings (18,22).

Healthcare Provider Motivation

Healthcare staff's intrinsic motivation and professional commitment were repeatedly highlighted as critical facilitators. Despite limited resources, providers expressed a sense of duty and perseverance: "What keeps us going is our motivation and the support of the community"(19). This motivation played a crucial role in maintaining service continuity under challenging conditions.

Education and Curriculum Inclusion

Positive developments in integrating palliative care into postgraduate and diploma-level curricula were noted. Certain academic programs now include palliative care components, providing a foundation for future capacity building: "Certain postgraduate and diploma programs include palliative care content" (21). Participants also called for reforming teaching methods to better match real-world practice.

Holistic Care Models

Specialized centers such as Hospice Ethiopia were cited for offering comprehensive, patient-centered care models that integrate medical, psychosocial, and spiritual services: "Hospice Ethiopia provided medical, psychosocial, financial, and spiritual support" (22). These models serve as benchmarks for scaling up integrated palliative care.

Stakeholder Involvement

Stakeholder recognition of the importance of palliative care and growing engagement from NGOs and religious institutions were seen as facilitators. "Some local and faith-based groups provide care and support at the grassroots level," noted one participant(18). Additionally, there is an increasing policy momentum: "We need stakeholders who understand the importance of palliative care"(20).

Confidence in Review Findings (GRADE-CERQual Assessment)

To evaluate the confidence in each synthesized theme, we applied the GRADE-CERQual (Confidence in the Evidence from Reviews of Qualitative Research) approach. This involved assessing each key review finding across four domains: (1) methodological limitations of the contributing studies, (2) coherence of the data supporting the finding, (3) adequacy of the data in terms of richness and quantity, and (4) relevance to the review question. Each theme was then

assigned an overall level of confidence: high, moderate, low, or very low. The rationale for each judgment was documented to ensure transparency and support the review's conclusions (Table 5). Among the ten themes identified, four were assessed with high confidence, including Health System Challenges, Sociocultural and Economic Factors, Community and Family Support, and Holistic Models of Care. These findings were consistently supported by rich, relevant data from multiple well-conducted studies. Five themes, such as Policy and Governance Gaps, Knowledge and Training Gaps, Collaboration Challenges, Healthcare Staff Motivation, and Stakeholder Involvement, were graded as having moderate confidence, largely due to some variability in perspectives or limited diversity among participants in certain studies. No themes were judged as low or very low confidence. This assessment strengthens the credibility of the findings and highlights where future research should focus to address remaining uncertainties — particularly in areas related to policy engagement, inter-organizational collaboration, and training reform.

Discussion

This is the first qualitative review in Ethiopia on palliative care services that identified five major barriers to effective palliative care implementation: policy and governance gaps, health system challenges, knowledge and training deficits, sociocultural and economic barriers, and collaboration challenges. This review found that policy and governance gaps are a major barrier to the implementation of palliative care in Ethiopia. This theme comprises four subthemes: absence of a national policy, limited stakeholder engagement, lack of a dedicated budget, and weak leadership and implementation frameworks. These challenges are not unique to Ethiopia, as similar barriers have been reported across various countries in low and Middle-Income Countries (23), Ghana(24), and Central and Eastern Europe (25) identified the absence of comprehensive national guidelines, insufficient integration of palliative care into existing health systems, and limited financial support as key obstacles to effective palliative care delivery. These parallels underscore the widespread nature of policy and governance issues hindering palliative care across various countries.

The absence of a formal national palliative care policy in Ethiopia has been identified as a significant structural barrier to effective palliative care delivery. This issue mirrors challenges observed in other low- and middle-income countries(23), where palliative care services are often not integrated into national health systems as studies done in India(26) and Egypt(27). The study emphasizes the necessity of Ethiopia to establish a comprehensive national palliative care policy, which should integrate palliative care into all healthcare systems, guarantee adequate professional

training, and secure sustainable funding. Addressing these gaps is essential to improve the quality of life for patients with life-limiting illnesses and to align Ethiopia's healthcare services with global standards for palliative care.

In Ethiopia, the limited engagement of stakeholders including community members, healthcare providers, and local leaders in the development of palliative care policies significantly hampers the relevance and effectiveness of these strategies. This exclusion leads to policies that are poorly understood and inadequately implemented, which is similar to the study done in Malawi(28). and Sub-Saharan Africa (29). These findings collectively highlight the critical importance of inclusive stakeholder engagement in the development and implementation of palliative care policies. Ensuring that all relevant parties are actively involved can lead to more effective, culturally appropriate, and sustainable palliative care services and improve health outcomes.

Many studies indicated that community members, healthcare providers, and local leaders are often not involved in policymaking, which limits the relevance and feasibility of the resulting strategies., This is similar to the study done in Kenya and Uganda(30). The Ethiopian context reflects this, where stakeholders at the implementation level feel disconnected from strategic planning.

Another significant challenge identified was the lack of a dedicated budget for palliative care, even the best-designed policies remain unimplemented, which aligns with findings from a multi-country analysis in the USA(31), which highlighted that palliative care services in Africa are frequently underfunded and dependent on external donor support, jeopardizing their sustainability. Ethiopian healthcare professionals in this review expressed similar concerns.

Finally, weak leadership and the absence of structured implementation mechanisms were cited as barriers to the scale-up of palliative care, which is similar to studies done in Norway(32), Israel(33), United States (34). They highlight the need for dedicated leadership, comprehensive training, and the integration of palliative care into organizational policies to overcome existing barriers and improve care delivery.

Health system challenges in Ethiopia emerged as another critical theme, including a shortage of trained staff, poor service integration, weak referral systems, urban-rural disparities, and inadequate infrastructure, which significantly hinder the delivery of palliative care. Many healthcare providers lack proper training in palliative care, which affects the quality of pain management and holistic support(18). Service integration remains weak, with fragmented care and ineffective referral systems, limiting continuity of care (19). Urban-rural disparities also persist,

as rural populations often lack access to palliative services due to geographic and infrastructure challenges(18). Inadequate medical infrastructure, including shortages in essential medications and equipment, further exacerbates the situation(19). These challenges are not isolated to Ethiopia, with similar findings in other sub-Saharan African countries, where health systems face difficulties in providing adequate palliative care, often due to underfunding, insufficient staffing, and poor service coordination(23). Addressing these barriers requires comprehensive reforms, including policy changes, improved healthcare workforce training, and better resource allocation to ensure equitable access to palliative care across all regions.

Knowledge and training deficits among healthcare professionals in Ethiopia remain major barriers to effective palliative care delivery(18,21). Many providers lack adequate training and express uncertainty in delivering comprehensive care, largely due to the absence of structured palliative care education in both undergraduate and in-service training programs, which is similar to a study done in Ethiopia (35). The limited and inconsistent integration of palliative care into curricula often reliant on individual instructors further exacerbates this gap. These findings highlight the urgent need for curriculum reform and standardized training to build a competent palliative care workforce.

Sociocultural and economic barriers become another theme that significantly limits the utilization of palliative care services in Ethiopia (18,22), consistent with findings from Ghana (36), Iran(37), and other low and middle-income countries(23). Cultural beliefs, financial constraints, and traditional healer preferences can hinder access to formal palliative care, leading to alternative treatments and exclusion from national health insurance schemes. These factors underscore the need for culturally sensitive, economically accessible, and integrated palliative care strategies.

Collaboration challenges between governments and non-governmental organizations significantly hinder the integration and delivery of palliative care services in Ethiopia(18), which is similar to a study done in Zimbabwe(38), India(39), and other low and middle-income countries(40). The global delivery and expansion of palliative care services are hindered by limited coordination between governments and NGOs due to policy differences and lack of integration into national health frameworks. Despite successful NGO-led models, sustainability remains a challenge without government support, highlighting the need for stronger policy-based collaboration.

Community and Family Support themes were most commonly reported from factors facilitating palliative care service utilization in Ethiopia (17,18,22). The study highlights the importance of community involvement, religious networks, and informal caregiving systems in sustaining care delivery, especially in rural and underserved settings, where formal mechanisms may not be available, which is a similar finding in a study done in the Netherlands(41), Colombia(42), India(43,44). Strengthening community-based approaches and promoting active family participation can enhance accessibility, sustainability, and quality of services, especially in underserved areas, and should be prioritized in global palliative care models.

Healthcare staff's intrinsic motivation and professional commitment were repeatedly highlighted as critical facilitators in palliative care utilization (19), which is a similar finding to a study done in Ghana(45), and East Africa (46). These examples underscore the importance of addressing healthcare provider motivation through both financial and non-financial means to improve healthcare delivery in Ethiopia and similar contexts.

Integrating palliative care into educational curricula is essential for building a competent healthcare workforce capable of addressing the comprehensive needs of patients with life-limiting illnesses(21). In Ethiopia, significant strides have been made in incorporating palliative care content into postgraduate and diploma-level programs(19), which was similarly highlighted in a study done in the Netherlands(41), Colombia(42), and India(43,44). Ethiopia's health education system struggles with palliative care integration due to limited presence in academic programs. Leaders focus on diploma and postgraduate courses, requiring curriculum reforms to align palliative care education with real-world clinical practices.

Holistic care models, exemplified by institutions like Hospice Ethiopia, are pivotal in delivering comprehensive palliative care that addresses the multifaceted needs of patients through the integration of medical, psychosocial, and spiritual services, ensuring patient-centered care that resonates with cultural and individual values(22). The finding is similar with study done in Uganda(47),India (48), Africa(49), Australia(50). These international examples underscore the importance of integrating holistic care models into palliative care services. By addressing the medical, psychosocial, and spiritual needs of patients, these models ensure comprehensive care that enhances the quality of life for individuals facing serious illnesses.

Stakeholder involvement is a crucial facilitator in advancing palliative care in Ethiopia, with growing engagement from NGOs, religious institutions, and policymakers(18,20). These

stakeholders contribute to service delivery, raise awareness, and provide support, particularly in rural and underserved areas. Similar trends are seen globally: in India, NGOs and religious institutions play a key role in expanding palliative care services, especially in rural areas(48), In the UK, strong policy backing and multi-sectorial collaboration have been instrumental in integrating palliative care into national healthcare systems (34). These international examples underscore the importance of stakeholder involvement in enhancing the reach and sustainability of palliative care services, ensuring comprehensive and holistic care for patients with life-limiting illnesses.

Strength and Limitation

A major strength of this review lies in its adherence to established methodological frameworks, including PRISMA and GRADE-CERQual, which ensured transparency, rigor, and credibility in synthesis and appraisal. Furthermore, the inclusion of diverse Ethiopian contexts enhanced the transferability of findings across regional and health facility settings. Based on these insights, the study recommends strengthening national palliative care policies, expanding training for healthcare providers, and promoting community awareness initiatives to address misconceptions and improve early utilization. Future research should consider longitudinal and implementation studies to evaluate the impact of targeted interventions on service uptake and patient outcomes.

Conclusion

This qualitative systematic review synthesized evidence from six studies to explore the multifaceted barriers and facilitators influencing palliative care utilization in Ethiopia. The findings revealed that sociocultural perceptions, limited awareness, inadequate healthcare infrastructure, lack of policy support, and workforce constraints significantly hinder access to palliative care, while strong familial support, community-based interventions, and integration into existing health systems emerged as key facilitators.

Declaration

Author contribution statement: The development and composition of this page have been greatly aided by SA and FK. Tables **1-5** and Figure **1** were prepared by SA and FK. Finally, all authors read & approved the manuscript for submission.

Competing interest statement: no conflict of interest.

Additional information: Not available.

Availability of data and materials

The datasets used and/or analyzed during the current study are available from the corresponding author upon reasonable request.

Ethics approval and consent to participate

Consent for publication

Not applicable.

Funding. No Funding.

Clinical trial number: CRD420251027739.

Human Ethics and Consent to Participate declarations: Not applicable

References

1. Radbruch L, Lima LD, Knaul F, Wenk R, Ali Z, Bhatnaghar S, et al. Redefining Palliative Care—A New Consensus-Based Definition. Journal of Pain and Symptom Management [Internet]. 2020 Oct 1;60(4). Available from: https://hsrc.himmelfarb.gwu.edu/smhs_medicine_facpubs/5119

2. Goh C (Cynthia), Wee B (Bee), Connor S (Stephen), Leng M (Mhoira), Pettus K (k), Moine S (Sébastien), et al. Redefining palliative care-a new consensus-based definition. 2020 [cited 2025 Apr 13]; Available from: https://hdl.handle.net/10171/67672

3. Radbruch L, Lima LD, Knaul F, Wenk R, Ali Z, Bhatnaghar S, et al. Redefining Palliative Care—A New Consensus-Based Definition. Journal of Pain and Symptom Management [Internet]. 2020 Oct 1;60(4). Available from: https://hsrc.himmelfarb.gwu.edu/smhs_medicine_facpubs/5119

4. Palliative Care Definition - IAHPC [Internet]. 2021 [cited 2025 Apr 13]. Available from: https://iahpc.org/research/consensus-based-definition-of-palliative-care/definition/

5. WHO Palliative care. WHO takes steps to address the glaring shortage of quality palliative care services [Internet]. 2021 [cited 2025 Apr 13]. Available from: https://www.who.int/news/item/05-10-2021-who-takes-steps-to-address-glaring-shortage-of-quality-palliative-care-services?utm_source=chatgpt.com

6. Connor SR, Centeno C, Garralda E, Clelland D, Clark D. Estimating the Number of Patients Receiving Specialized Palliative Care Globally in 2017. J Pain Symptom Manage. 2021 Apr;61(4):812–6.

7. Sleeman KE, Brito M de, Etkind S, Nkhoma K, Guo P, Higginson IJ, et al. The escalating global burden of serious health-related suffering: projections to 2060 by world regions, age groups, and health conditions. The Lancet Global Health. 2019 Jul 1;7(7):e883–92.

8. WHO Fact Sheet on Palliative Care. WHO takes steps to address the glaring shortage of quality palliative care services [Internet]. 2022 [cited 2025 Apr 13].

9. Knaul FM, Bhadelia A, Rodriguez NM, Arreola-Ornelas H, Zimmermann C. The Lancet Commission on Palliative Care and Pain Relief—findings, recommendations, and future directions. The Lancet Global Health. 2018 Mar 1;6:S5–6.

10. Sallnow L, Smith R, Ahmedzai SH, Bhadelia A, Chamberlain C, Cong Y, et al. Report of the Lancet Commission on the Value of Death: bringing death back into life. Lancet. 2022;399(10327):837–84.

11. Clark D, Baur N, Clelland D, Garralda E, López-Fidalgo J, Connor S, et al. Mapping Levels of Palliative Care Development in 198 Countries: The Situation in 2017. J Pain Symptom Manage. 2020 Apr;59(4):794-807.e4.

12. Moher D, Liberati A, Tetzlaff J, Altman DG. Preferred Reporting Items for Systematic Reviews and Meta-Analyses: The PRISMA Statement. Journal of Clinical Epidemiology. 2009 Oct 1;62(10):1006–12.

13. Tong A, Flemming K, McInnes E, Oliver S, Craig J. Enhancing transparency in reporting the synthesis of qualitative research: ENTREQ. BMC Med Res Methodol. 2012 Nov 27;12:181.

14. JBI Manual for Evidence Synthesis. JBI Manual for Evidence Synthesis - JBI Global Wiki [Internet]. 2024 [cited 2025 Apr 12]. Available from: https://jbi-global-wiki.refined.site/space/MANUAL

15. Singh J. Critical appraisal skills program. Journal of Pharmacology and Pharmacotherapeutics. 2013 Mar 1;4(1):76–7.

16. Thomas J, Harden A. Methods for the thematic synthesis of qualitative research in systematic reviews. BMC Med Res Methodol. 2008 Jul 10;8:45.

17. Wainwright M, Zahroh RI, Tunçalp Ö, Booth A, Bohren MA, Noyes J, et al. The use of GRADE-CERQual in qualitative evidence synthesis: an evaluation of fidelity and reporting. Health Research Policy and Systems. 2023 Jul 25;21(1):77.

18. Abate Y, Solomon K, Azmera YM, de Fouw M, Kaba M. Barrier analysis for continuity of palliative care from health facility to household among adult cancer patients in Addis Ababa, Ethiopia. BMC Palliat Care. 2023 May 12;22(1):57.
19. Negasa E, Human SP, Roro A. Challenges in Palliative Care Provision in Ethiopia: An Exploratory Qualitative Study. JPR. 2023 Oct;Volume 16:3405–15.
20. Aregay A, O’Connor M, Stow J, Ayers N, Lee S. Palliative care in Ethiopia’s rural and regional health care settings: a qualitative study of enabling factors and implementation challenges. BMC Palliat Care. 2023 Oct 17;22(1):156.
21. Aregay A, O’Connor M, Stow J, Ayers N, Lee S. Perceived policy-related barriers to palliative care implementation: a qualitative descriptive study. Palliat�Care. 2023 Jan;17:26323524231198542.

22. Aregay A, O’Connor M, Stow J, Ayers N, Lee S. Measuring and exploring the barriers to translating palliative care knowledge into clinical practice in rural and regional health-care settings. Palliative & Supportive Care. 2024;22(6):1605–14.
23. Kaba M, de Fouw M, Deribe KS, Abathun E, Peters AAW, Beltman JJ. Palliative care needs and preferences of female patients and their caregivers in Ethiopia: A rapid program evaluation in Addis Ababa and Sidama zone. PLoS One. 2021;16(4):e0248738.

24. Hannon B, Zimmermann C, Knaul FM, Powell RA, Mwangi-Powell FN, Rodin G. Provision of Palliative Care in Low- and Middle-Income Countries: Overcoming Obstacles for Effective Treatment Delivery. JCO. 2016 Jan;34(1):62–8.
25. Okyere J, Kissah-Korsah K. Barriers to the integration of palliative care in Ghana: evidence from a tertiary health facility. Palliat Care Soc Pract. 2023 Jun 14;17:26323524231179980.

26. Lynch T, Clark D, Centeno C, Rocafort J, Flores LA, Greenwood A, et al. Barriers to the Development of Palliative Care in the Countries of Central and Eastern Europe and the Commonwealth of Independent States. Journal of Pain and Symptom Management. 2009 Mar 1;37(3):305–15.

27. palliumindia. Pallium India. 2012 [cited 2025 Apr 13]. Government of India's Palliative Care Strategy. Available from: https://palliumindia.org/2012/11/government-of-indias-palliative-care-strategy

28. Ibrahim A, Abdelmeged R, El-Sakhawy M, Zaghamir D, Elsehrawey M, Elalem O, et al. Palliative and Supportive Care for Patients with End-Stage Hepatocellular Carcinoma: Perspectives of Egyptian Domestic Caregivers. Asian Pac J Cancer Prev. 2023 Dec 1;24(12):4179–86.

29. Karuga R, Kok M, Luitjens M, Mbindyo P, Broerse JEW, Dieleman M. Participation in primary health care through community-level health committees in Sub-Saharan Africa: a qualitative synthesis. BMC Public Health. 2022 Feb 19;22(1):359.
30. Karuga R, Kok M, Luitjens M, Mbindyo P, Broerse JEW, Dieleman M. Participation in primary health care through community-level health committees in Sub-Saharan Africa: a qualitative synthesis. BMC Public Health. 2022 Feb 19;22(1):359.

31. Fraser BA, Powell RA, Mwangi-Powell FN, Namisango E, Hannon B, Zimmermann C, et al. Palliative Care Development in Africa: Lessons From Uganda and Kenya. J Glob Oncol. 2018 Sep;4:1–10.

32. Knaul FM, Farmer PE, Krakauer EL, De Lima L, Bhadelia A, Jiang Kwete X, et al. Alleviating the access abyss in palliative care and pain relief imperative of universal health coverage: the Lancet Commission report. Lancet. 2018 Apr 7;391(10128):1391–454.

33. Sommerbakk R, Haugen DF, Tjora A, Kaasa S, Hjermstad MJ. Barriers to and facilitators for implementing quality improvements in palliative care - results from a qualitative interview study in Norway. BMC Palliat Care. 2016 Jul 15;15:61.

34. Dakka FJ. Nurses Barriers to Evidence-Based Practice in Palliative Care: A Systematic Review. SAGE Open Nurs. 2022 Dec 9;8:23779608221142957.

35. Aldridge MD, Hasselaar J, Garralda E, van der Eerden M, Stevenson D, McKendrick K, et al. Education, implementation, and policy barriers to greater integration of palliative care: A literature review. Palliat Med. 2016 Mar;30(3):224–39.

36. Wake AD. Knowledge and associated factors towards palliative care among nurses in Ethiopia: A systematic review and meta-analysis. SAGE Open Med. 2022;10:20503121221092338.

37. Okyere J, Kissah-Korsah K. Barriers to the integration of palliative care in Ghana: evidence from a tertiary health facility. Palliat Care Soc Pract. 2023;17:26323524231179980.

38. Hassankhani H, Rahmani A, Best A, Taleghani F, Sanaat Z, Dehghannezhad J. Barriers to home-based palliative care in people with cancer: A qualitative study of the perspective of caregivers. Nurs Open. 2020 Apr 22;7(4):1260–8.

39. Magocha B, Molope M, Palamuleni M, Saruchera M. The Interface between the State and NGOs in Delivering Health Services in Zimbabwe—A Case of the MSF ART Programme. International Journal of Environmental Research and Public Health. 2023 Jan;20(23):7137.

40. Krishnan A, Rajagopal MR, Karim S, Sullivan R, Booth CM. Palliative Care Program Development in a Low- to Middle-Income Country: Delivery of Care by a Nongovernmental Organization in India. JGO. 2018 Mar;(4):1–8.
41. Hannon B, Zimmermann C, Knaul FM, Powell RA, Mwangi-Powell FN, Rodin G. Provision of Palliative Care in Low- and Middle-Income Countries: Overcoming Obstacles for Effective Treatment Delivery. JCO. 2016 Jan;34(1):62–8.
42. Schutter T, Koper I, Vissers K, Hasselaar J. Social support sources for people with palliative care needs at home: a scoping review. BMC Palliative Care. 2025 Feb 4;24(1):34.

43. Vargas-Escobar LM, Hernández-Rincón EH, León-Delgado MX, Muñoz-Medina SE, Mantilla-Manoslava N, Correa-Morales JE, et al. Enhancing rural community engagement through palliative care networks: A scoping review. Health Policy. 2024 Nov 1;149:105165.

44. Gupta M, Kankaria A, Joshy LE, Singh S, Lal B, Choudhary S, et al. Community-based palliative care needs and barriers to access among cancer patients in rural north India: Participatory action research. BMC Palliative Care. 2024 Oct 9;23(1):240.

45. Fereydooni S, Lorenz KA, Ganesh A, Satija A, Spruijt O, Bhatnagar S, et al. Empowering families to take on a palliative caregiver role for patients with cancer in India: Persistent challenges and promising strategies. PLOS ONE. 2022 Sep 16;17(9):e0274770.

46. Alhassan RK, Spieker N, van Ostenberg P, Ogink A, Nketiah-Amponsah E, de Wit TFR. Association between health worker motivation and healthcare quality efforts in Ghana. Hum Resour Health. 2013 Aug 14;11:37.
47. Muthuri RNDK, Senkubuge F, Hongoro C. Determinants of Motivation among Healthcare Workers in the East African Community between 2009-2019: A Systematic Review. Healthcare (Basel). 2020 Jun 10;8(2):164.
48. Holistic Care – Hospice Africa. Holistic Care – Hospice Africa [Internet]. 2025 [cited 2025 Apr 13]. Available from: https://www.hospice-africa.org/holistic-care/

49. Singh T, Harding R. Palliative care in South Asia: a systematic review of the evidence for care models, interventions, and outcomes. BMC Res Notes. 2015 Dec;8(1):1–15.
50. Clark D, Wright M, Hunt J, Lynch T. Hospice and Palliative Care Development in Africa: A Multi-Method Review of Services and Experiences. Journal of Pain and Symptom Management. 2007 Jun 1;33(6):698–710.

51. Laabar TD, Saunders C, Auret K, Johnson CE. Socially, Culturally and Spiritually Sensitive Public Health Palliative Care Models in the Lower-income Countries: An Integrative Literature Review. Indian J Palliat Care. 2023;29(1):15–27.

Click here to access/download
Supporting Information
List of Tables.docx

Click here to access/download
Supporting Information
PRISMA_2020_Checklist_Qualitative.docx
