# Supplementary material for: Barriers and facilitators to palliative care service utilization in Ethiopia: A qualitative systematic review, 2025
Source: PLoS One. 2025 Aug 4;20(8):e0328222. doi: 10.1371/journal.pone.0328222 (PMC12321145; doi:10.1371/journal.pone.0328222)
Supplement: Attachment — Submitted filename: Response to Reviewers.pdf [file pone.0328222.s005.pdf]

Response to the Editor and reviewers

Response to the Editor

Thank you very much for your valuable suggestions and continued support throughout the revision process. We are grateful for your insightful comments, which have significantly contributed to improving the quality and readability of our manuscript.

# 1. Regarding the focus and length of the manuscript, we have carefully reviewed the text and made efforts to shorten and clarify sections to improve conciseness and readability, ensuring the key messages are clear and focused.

#2. For the references, we have thoroughly checked and corrected all citations, including reformatting reference 2, removing any undefined characters from reference 27, adjusting journal abbreviations in reference 12, aligning reference 19 with the updated text, and adding access dates where applicable for internet sources. We ensured all references now conform to the journal's citation style and standards.

We appreciate your guidance, which has helped improve the manuscript's clarity and quality.
Thank you once again for your guidance and consideration

Response to Reviewer #1

Thank you very much for your encouraging feedback and for recognizing the relevance and importance of our study. We truly appreciate your thoughtful suggestion regarding potential improvements in presentation. In response, we have carefully reviewed and refined the manuscript to enhance clarity, coherence, and readability throughout. We are grateful for your recommendation and your support for the publication of this work, which aims to bring greater visibility to a critical yet under-addressed area in health systems and scientific discourse.

Response to Reviewer #2

Thank you for your insightful feedback and helpful suggestion regarding the structure of the Discussion section. In response, we have revised the section to adopt a more synthesized approach, as recommended. We believe this reorganization improves clarity and enhances the comparative value of our findings.

#1. **Reviewer Comment -Overall comment on discussion-**The discussion details theme by theme and even sub-theme from the results attempting to contrast them against existing literature.

Author Response to Reviewer #2 – Overall Comment on Discussion Section:

Thank you very much for your constructive feedback regarding the organization of the Discussion section. We agree with your observation that a theme-by-theme approach may create unnecessary complexity and reduce clarity for readers. In response to your valuable suggestion, we have revised the Discussion section to present a more synthesized narrative. Specifically:

1. We now begin by **jointly discussing themes that align with global or regional experiences**, such as policy gaps, national palliative care strategies, and implementation frameworks. This comparative synthesis helps place the findings within a broader context.
2. Subsequently, we highlight and elaborate on **themes uniquely relevant to the Ethiopian context**, such as sociocultural norms, religious influences, the role of Hospice Ethiopia, and locally driven training initiatives. This restructuring allows the reader to clearly distinguish between universally shared challenges and those specific to Ethiopia.

   We believe this new format improves the overall coherence and readability of the Discussion and better reflects the significance of our findings.

#2. **Reviewer Comment (Page 4, Paragraph "Many countries still lack…..and increased health costs"):**This section should be reformulated in a more concise way. Currently it jumps from root causes to consequences to recommendations, then back to causes followed by consequences. The flow of ideas could be improved by sticking to cause–consequence structure.

Author Response:

Thank you for this valuable feedback. We agree that the paragraph's structure required improvement for better clarity and logical flow. Accordingly, we have revised the section to follow a clear cause–consequence structure. The updated version now presents the lack of policies, medications, trained personnel, and care models as root causes, followed by the resulting consequences for patients and health systems. We believe this change enhances the coherence and readability of the text. The revised paragraph can be found on Page 4, Paragraph 2 of the revised manuscript. <u>Many countries still lack national policies, access to essential medications, trained professionals, and community-based care models, resulting in widespread suffering and poor quality of life for patients with serious illnesses (5–8).</u>

<u>The consequences are profound: millions die each year in severe pain and distress, reflecting a global health system failure and raising serious ethical and human rights concerns. Addressing</u>

this crisis requires urgent action to integrate palliative care into universal health coverage and strengthen healthcare systems using culturally appropriate approaches (9).

#3. Reviewer Comment:
"Please insert a reference for the 'SPIDER tool.'"

Author Response:

Thank you for your helpful suggestion. We have now inserted an appropriate reference to support the use of the SPIDER tool in qualitative evidence synthesis. The reference we included is:Cooke, A., Smith, D., & Booth, A. (2012). Beyond PICO: The SPIDER tool for qualitative evidence synthesis. Qualitative Health Research, 22(10), 1435–1443.

https://doi.org/10.1177/1049732312452938.
This has been added in the Methods section where the SPIDER tool is first mentioned.

#4. **Reviewer Comment (Page 9, Line 16):**
You seem to be missing a title for this section "Theme formulated as Barriers to Palliative Care"? Later in the text you use a title for the "Facilitators". It needs to be consistent.

Author Response:

Thank you for your helpful observation. We agree that the section required a clear and consistent heading to match the format used for the facilitators. Accordingly, we have added the title "Barriers to the Implementation and Delivery of Palliative Care" at the beginning of the section (Page 9, Line 16) to improve clarity and maintain structural consistency throughout the synthesis. Then we add Theme formulated as Barriers to Palliative Care for barriers theme.

#5. Reviewer Comment (Page 9, Line 21):
"In Ethiopia, cultural and religious norms significantly influence end-of-life care preferences,…. culturally sensitive palliative care models." While this has been added in the revision on the editor's request, the paragraph's position in the discussion is odd. I would suggest moving this to page 13 where you discuss engagement with community, local leader and cultural appropriateness. Or to page 15 where you discuss "Socio-cultural and economic barriers".

Author Response:
Thank you for this insightful suggestion. We agree that the paragraph discussing cultural and religious influences on end-of-life care fits more appropriately within the section on "Socio-cultural and economic barriers." Accordingly, we have moved the paragraph from Page 9 to Page 15 to ensure better thematic alignment and narrative flow. We believe this relocation strengthens the coherence of the discussion and better supports the interpretation of the findings.

#6 Reviewer Comment (Page 12, Line 29):
"These challenges are not unique to Ethiopia, as similar barriers have been reported across various countries in low and Middle-Income Countries." Please revise as "These challenges are

not unique to Ethiopia, as similar barriers have been reported across various LMICs" to avoid repetition.

Author Response:

Thank you for the helpful suggestion. We have revised the sentence accordingly to improve *conciseness and avoid repetition. The sentence now reads: "These challenges are not unique to Ethiopia, as similar barriers have been reported across various LMICs." This change has been made on Page 12, Line 29 of the revised manuscript.*

#7 Reviewer Comment (Page 13, Line 5):

Please replace "low- and middle-income countries" with the acronym throughout the text, as it is repeated many times.

Author Response:

Thank you for this helpful suggestion. We have replaced all instances of "low- and middle-income countries" with the acronym "LMICs" throughout the manuscript to improve readability and reduce repetition.

#8 Reviewer Comment (Page 13, Line 6):

"The study emphasizes the necessity of Ethiopia…"

Unclear to what "the study" refers to—is it the current manuscript or another paper cited?

Author Response:

Thank you for pointing this out. To improve clarity, we have revised the sentence to explicitly indicate that it refers to the current study. The sentence now reads: ***"Findings from this study emphasize the necessity for Ethiopia to establish a comprehensive national palliative care policy…"*** *This change has been made on Page 13, Line 6 of the revised manuscript.*

#9 Reviewer Comment (Page 13, Line 24):

"You seem to be referencing the Lancet Commission paper on palliative care as 'a multi-country analysis in the USA'. Please revise."

Author Response:

Thank you for highlighting this important point. We have corrected the description to accurately reflect the source. The revised sentence now reads: ***"Another significant challenge identified was the lack of a dedicated budget for palliative care. Even the best-designed policies remain unimplemented, which aligns with findings from the Lancet Commission on Global Access to Palliative Care and Pain Relief (37), which highlighted that palliative care services in Africa are frequently underfunded and reliant on external donor support, jeopardizing their sustainability."*** *This correction has been made on Page 13, Line 24 of the revised manuscript. The full reference has also been updated in the reference list as follows:* ***Knaul FM, Farmer PE, Krakauer EL, et al. (2018).*** *Alleviating the access abyss in palliative*

*care and pain relief—an imperative of universal health coverage: the Lancet Commission report. The Lancet, **391**(10128), 1391–1454. https://doi.org/10.1016/S0140-6736(17)32513-8*

#10. Reviewer Comment (Page 15, Line 23):
"In Ethiopia, significant strides have been made in incorporating palliative care content into postgraduate and diploma-level programs….. Ethiopia's health education system struggles with palliative care integration due to limited presence in academic programs….."
This is unclear; you seem to be arguing one thing and the opposite at the same time. I'd suggest rephrasing.

Author Response:
Thank you for your valuable observation. We agree that the original paragraph presented a contradiction. To clarify, we have revised the text to acknowledge that while some progress has been made at the postgraduate and diploma levels, overall integration of palliative care into the broader health education system remains limited. The revised version improves consistency and better reflects the current state of palliative care education in Ethiopia. This change has been made on Page 15, Line 23 of the revised manuscript.

We hope that our revisions adequately address your concerns and improve the quality of the manuscript. We sincerely appreciate your thoughtful review and helpful suggestions, which have strengthened our work. Thank you again for your consideration.

Response for review #3

Thank you very much for your thorough and constructive feedback on our manuscript. We greatly appreciate the time and effort you have taken to provide valuable insights. Please find below our detailed responses to each of your comments and the corresponding revisions made in the manuscript.

#1. Reviewer comment:
"The search strategy lacks transparency. There is no annex or appendix with the exact search equation used. For the sake of reproducibility and clarity, it would be important to include the complete search string(s) for each database."

Author response:

*Thank you for this valuable comment. To improve transparency and reproducibility, we have now included the complete search strategies used for each database in a new **Supporting Information file (S1 Appendix)**, as recommended by PLOS ONE guidelines. In the revised manuscript, we have added a reference to this appendix in the Search Strategy section of the Methods. The detailed search strings for PubMed/MEDLINE, Scopus, Web of Science, CINAHL, and Google Scholar are provided in this appendix.*

#2. Reviewer comment:
"It appears that the review was registered in PROSPERO after the data search and screening process. This raises concerns regarding adherence to best practices for systematic review methodology. Please explain the rationale for the delayed registration."

Author response:

Thank you for this important observation. We acknowledge that the PROSPERO registration (CRD420251027739) was completed on April 6, 2025, shortly after the initial data search and screening had begun. This delay was due to the time required to finalize the review protocol to ensure it fully complied with PROSPERO's detailed submission requirements and the Joanna Briggs Institute (JBI) standards.

Despite the timing of registration, the review protocol was developed in advance and strictly followed throughout the review process. The protocol pre-specified the review objectives, eligibility criteria, search strategy, data extraction, quality appraisal, and synthesis methods, ensuring transparency and minimizing the risk of bias. We have clarified this timeline and process in the revised manuscript to assure adherence to systematic review best practices.

#3. Reviewer comment:
"The section on 'handling of missing data' is not appropriate for a qualitative systematic review, as such reviews do not involve analysis of raw quantitative data. Consider removing this section, as discussions on missing data are relevant primarily to quantitative systematic reviews."

Author response:

Thank you for this helpful comment. We agree that the discussion on "handling of missing data" is more relevant to quantitative systematic reviews and does not apply to our qualitative synthesis. In response, we have removed the original section and replaced it with a more appropriate and concise statement on data completeness, which affirms that all included studies provided sufficient information for thematic analysis and that no reporting gaps affected the synthesis. This change aligns better with qualitative review methodology while maintaining transparency.

#4. Reviewer comment:

"Figure 1 – PRISMA Flow Diagram: Consider using the official PRISMA 2020 flow diagram template, available on the PRISMA website, for consistency and clarity."

Author response:

Thank you for this constructive suggestion. In response, we have revised Figure 1 using the official ***PRISMA 2020 flow diagram template****, as recommended. This ensures consistency with current reporting standards and improves the clarity of the study selection process. The updated figure has been included in the revised manuscript.*

#5. Reviewer comment:

"Table 1 – Formatting and Style"-The font type and size used in Table 1 is inconsistent with the rest of the manuscript. Some article titles appear in bold while others do not. Please ensure consistent formatting throughout the table and match the main text style

Author Response:

Thank you for your valuable feedback. We have carefully revised Table 1 to ensure consistent font type and size throughout the table, aligning it with the main manuscript text style. All article titles are now uniformly formatted without bold text, and the overall presentation has been standardized to improve readability. The updated table is included in the revised manuscript.

#6. Reviewer comment:

"The formatting of Table 2 needs improvement. The text is cut off, and the column layout is difficult to read. Please revise the table to enhance legibility and alignment."

Author response:

Thank you for this helpful feedback. We have revised Table 2 to improve formatting, text alignment, and column layout. The updated table ensures that all content is fully visible and clearly organized for better readability. The revised version has been included in the updated manuscript.

We hope that our revisions adequately address your concerns and improve the quality of the manuscript. We sincerely appreciate your thoughtful review and helpful suggestions, which have strengthened our work. Please do not hesitate to contact us if you require any further clarification.

Thank you again for your consideration.
