# Supplementary material for: Barriers and facilitators to palliative care service utilization in Ethiopia: A qualitative systematic review, 2025
Source: PLoS One. 2025 Aug 4;20(8):e0328222. doi: 10.1371/journal.pone.0328222 (PMC12321145; doi:10.1371/journal.pone.0328222)
Supplement: Attachment — Submitted filename: Response to the editor and reviewers.docx [file pone.0328222.s004.docx]

# **Response to the editor and reviewers**

# **Response to Editor**

Thank you for your thoughtful feedback on our manuscript and for highlighting important areas for improvement. We appreciate your constructive suggestions and have addressed them as follows:

1. **Palliative care is not only about pain but also other symptoms (Page 5):**
   We agree with your observation. We have revised the paragraph to reflect the holistic nature of palliative care, which addresses not only pain but also a wide range of physical, psychological, social, and spiritual symptoms. This aligns with the broader definition of palliative care and strengthens the comprehensiveness of our discussion.
2. **Use of local references to describe the situation in Ethiopia (Page 5):**
   In response to your suggestion, we have incorporated several recent and relevant local studies to better contextualize the barriers to palliative care in Ethiopia. These references support our discussion on challenges related to medication availability, provider training, sociocultural perceptions, and system-level gaps.
3. **PRISMA-QES – Clarification of Status (Work in Progress)**

**Comment**: PRISMA-QES is a work in progress. You may wish to include that precision.

**Response**:
We appreciate this important note. We have added clarification that the PRISMA-QES is currently under development and evolving. This helps manage reader expectations about its application.

**Amendment**:
We inserted the following statement in the **Methods section (Page 6)**:

"Although PRISMA-QES (an extension for qualitative evidence synthesis) is used as a guide, it is important to note that it remains under development and is considered a work in progress."

4. **Cultural Preferences for Home-Based Death and Role of Religion**

**Comment**: The reader may want to understand more about cultural preferences for home-based death and the Ethiopian approach. What role do various religions play?

**Response**:
We have added content that outlines cultural values around home-based care and end-of-life preferences in Ethiopia, including the influence of major religions like Orthodox Christianity, Islam, and Protestantism.

**Amendment**:
We included this in the **Discussion section (Page 15)**:

"In Ethiopia, cultural and religious norms significantly influence end-of-life care preferences, with many patients and families favoring home-based care due to its alignment with their spiritual values and spiritual fulfillment, emphasizing the need for culturally sensitive palliative care models."

5. **Three of Six Studies from One Research Group – Risk of Bias**

**Comment**: Three of six studies come from one research group. This should be discussed.

**Response**:
We agree this could introduce potential bias. We have now discussed the implications of this concentration of studies on the interpretation of results.

**Amendment**:
We added the following to the **Limitations section (Page 19)**:

"It is worth noting that three out of the six included studies originated from the same research group, which may introduce bias and limit the diversity of perspectives. This potential imbalance should be considered when interpreting the results."

6. **Mixing High- and Low-Income Countries – Focus on Low-Income Settings**

**Comment**: Your discussion mixes high- and low-income countries. Focus on low-income countries. Also consider regional differences within Ethiopia.

**Response**:
We revised the discussion to emphasize findings from low-income countries, particularly Sub-Saharan Africa, and highlighted disparities between Ethiopian regions.

**Amendment**:
Changes were made in the **Discussion section (Page 14)**:

" This review focuses on low-income settings in Ethiopia, where regional disparities between urban centers and rural areas complicate equitable access to palliative care.."

7. **Extend Conclusion with Practical Proposals for Improvement**

**Comment**: Extend the conclusion with proposals on how to improve the situation.

**Response**:
We appreciate this suggestion and have added practical recommendations to improve palliative care access and quality in Ethiopia.

**Amendment**:
We revised the **Conclusion (Page 19)** to include:

"To address these barriers, we recommend strengthening policy integration of palliative care, expanding training programs for healthcare providers, improving access to essential medications, and promoting community awareness. Culturally sensitive home-based care models and faith-based collaborations should also be explored."

8. **Typographical Errors, Unexplained Abbreviations, and Reference Formatting**

**Comment**: There are several typographical errors, unexplained abbreviations, and inconsistencies in reference formatting.

**Response**:
We have carefully proofread the entire manuscript to correct typographical errors, ensured all abbreviations are defined on first use, and standardized the reference list in accordance with journal guidelines.

**Amendment**:

- All abbreviations were reviewed and explained at first mention
- Reference formatting was standardized and incomplete entries corrected
- Typographical errors were corrected throughout the text.

**We hope these revisions meet the expectations and improve the overall quality of the manuscript. We remain grateful for the detailed and constructive comments.**

Sincerely,
Sadik Abdulwehab

On behalf of all co-authors

# Response for review one

**Dear Reviewer,**

We sincerely thank you for your thoughtful and constructive feedback. We appreciate your recognition of our manuscript's contribution in presenting a comprehensive view of our mansuscript. Your comments have been invaluable in helping us improve the clarity, coherence, and overall quality of the manuscript. Below, we respond point by point to your main comment:

**1. Reflexivity Reporting (added under Quality Appraisal section):**

*"Although reflexivity was not consistently addressed in the included studies, we acknowledge its importance in qualitative research. Future studies should systematically report researcher reflexivity, including how their positions, assumptions, or professional roles may influence data interpretation. In our review process, we remained aware of our own professional backgrounds as nurse educators and researchers, and we critically reflected on how these roles may shape our thematic synthesis and interpretation of findings."*

**2. Sample Size and Transferability (added under Strength and Limitation section):**

*"However, the limited number of included studies (n=6) may affect the transferability of the findings, particularly to underrepresented regions or patient populations. Further research is needed to expand the evidence base and include perspectives from more varied sociocultural and geographic contexts."*

**3. Clarifying Data Availability (revised the Availability of Data and Materials section):**

*“The data supporting this review consist of synthesized qualitative findings extracted from publicly available primary studies. A complete data extraction table, detailing study characteristics and key findings, is included as a supplementary file to enhance transparency and facilitate verification."*

Again We are grateful for your detailed feedback, which has strengthened the manuscript.

# Response for review two

Dear Reviewer,

We sincerely thank you for your thoughtful and constructive feedback. We appreciate your recognition of our manuscript's contribution in presenting a comprehensive view of barriers and facilitating factors to palliative care provision in Ethiopia and other low- and middle-income countries (LMICs). Your comments have been invaluable in helping us improve the clarity, coherence, and overall quality of the manuscript. Below, we respond point by point to your main comment:

**Reviewer Comment:** *“The manuscript suffers from a lack of copy editing. Multiple sections are repeated a few sentences apart. I strongly invite the authors to substantially revise the manuscript deleting the repeated statements and merging some sections to help the flow of ideas. These are particularly prevalent in the introduction and results sections.”*

**Response:**

Thank you for this important observation. In response, we conducted a comprehensive revision of the manuscript, particularly focusing on the **Introduction** and **Results** sections as you advised.

- In the **Introduction**, we removed repetitive statistics and overlapping background information regarding global palliative care needs, especially those related to WHO data and LMIC comparisons. These were merged into a single, cohesive paragraph that communicates the global context more effectively and without redundancy.
- We also restructured the Ethiopian context section by combining fragmented sentences about limited access, urban-rural disparities, and policy challenges into a more fluid, coherent narrative. This improved readability and eliminated repetition.
- In the **Results** section, we removed redundant descriptions of study settings, participant diversity, and study objectives. We combined overlapping paragraphs to ensure that each study’s contribution was presented clearly and concisely.
- Additionally, we identified and eliminated a duplicated subsection titled “Policy and Governance Gaps” that had been inadvertently repeated.
- Throughout the manuscript, we carefully reviewed and revised each paragraph for improved clarity, conciseness, and flow to ensure the narrative is more streamlined and professional.

We believe these revisions have significantly improved the manuscript's structure, coherence, and readability. We are grateful for your detailed feedback, which has strengthened the manuscript considerably. Please let us know if there are any additional areas that require further clarification or improvement.

Sincerely,

Sadik Abdulwehab
